# Harnessing acoustic speech parameters to decipher amyloid status in individuals with mild cognitive impairment

**DOI:** 10.3389/fnins.2023.1221401

**Published:** 2023-09-07

**Authors:** Fernando García-Gutiérrez, Marta Marquié, Nathalia Muñoz, Montserrat Alegret, Amanda Cano, Itziar de Rojas, Pablo García-González, Clàudia Olivé, Raquel Puerta, Adelina Orellana, Laura Montrreal, Vanesa Pytel, Mario Ricciardi, Carla Zaldua, Peru Gabirondo, Wolfram Hinzen, Núria Lleonart, Ainhoa García-Sánchez, Lluís Tárraga, Agustín Ruiz, Mercè Boada, Sergi Valero

**Affiliations:** ^1^Ace Alzheimer Center Barcelona, Universitat Internacional de Catalunya, Barcelona, Spain; ^2^Networking Research Center on Neurodegenerative Diseases (CIBERNED), Instituto de Salud Carlos III, Madrid, Spain; ^3^Accexible Impacto s.l., Urduliz, Bizkaia, Spain; ^4^Department of Translation and Language Sciences, Universitat Pompeu Fabra, Barcelona, Spain; ^5^Institut Català de Recerca i Estudis Avançats (ICREA), Barcelona, Spain

**Keywords:** Alzheimer's disease, mild cognitive impairment, early diagnosis, cerebrospinal fluid, biomarkers, machine learning, speech acoustics, automated pattern recognition

## Abstract

Alzheimer's disease (AD) is a neurodegenerative condition characterized by a gradual decline in cognitive functions. Currently, there are no effective treatments for AD, underscoring the importance of identifying individuals in the preclinical stages of mild cognitive impairment (MCI) to enable early interventions. Among the neuropathological events associated with the onset of the disease is the accumulation of amyloid protein in the brain, which correlates with decreased levels of A*β*42 peptide in the cerebrospinal fluid (CSF). Consequently, the development of non-invasive, low-cost, and easy-to-administer proxies for detecting A*β*42 positivity in CSF becomes particularly valuable. A promising approach to achieve this is spontaneous speech analysis, which combined with machine learning (ML) techniques, has proven highly useful in AD. In this study, we examined the relationship between amyloid status in CSF and acoustic features derived from the description of the Cookie Theft picture in MCI patients from a memory clinic. The cohort consisted of fifty-two patients with MCI (mean age 73 years, 65% female, and 57% positive amyloid status). Eighty-eight acoustic parameters were extracted from voice recordings using the extended Geneva Minimalistic Acoustic Parameter Set (eGeMAPS), and several ML models were used to classify the amyloid status. Furthermore, interpretability techniques were employed to examine the influence of input variables on the determination of amyloid-positive status. The best model, based on acoustic variables, achieved an accuracy of 75% with an area under the curve (AUC) of 0.79 in the prediction of amyloid status evaluated by bootstrapping and Leave-One-Out Cross Validation (LOOCV), outperforming conventional neuropsychological tests (AUC = 0.66). Our results showed that the automated analysis of voice recordings derived from spontaneous speech tests offers valuable insights into AD biomarkers during the preclinical stages. These findings introduce novel possibilities for the use of digital biomarkers to identify subjects at high risk of developing AD.

## 1. Introduction

Alzheimer's disease (AD) stands as the primary contributor to dementia cases worldwide, with no effective treatment available (Alzheimer's & Dementia, 2023). This progressive neurodegenerative disease impacts different cognitive domains, including memory, language, attention, and behavior, ultimately incapacitating the individual from performing daily tasks (Alzheimer's & Dementia, 2023). The disease's pathophysiology involves the formation of amyloid-β plaques (Aβ) and neurofibrillary tangles of phosphorylated tau protein (p-tau) in the brain. The accumulation of these two substrates eventually leads to neuroinflammation, reduced brain metabolism, and atrophy, which underlie the observed cognitive alterations (Peña-Casanova et al., 2012). However, compelling evidence suggests that the pathophysiological events related to AD begin several years, even decades, before the onset of clinical symptoms (Sperling et al., 2011). Therefore, much of the efforts in the field have been focused on identifying individuals in the early stage of mild cognitive impairment (MCI) (Alzheimer's & Dementia, 2023).

Once an individual with AD has progressed to the dementia stage and there is a loss of autonomy, i.e., cognitive impairment is already evident, the opportunities for potential disease-modifying interventions become limited. Consequently, several AD diagnostic criteria recommend the use of biomarkers tightly associated with AD pathological hallmarks (McKhann et al., 2011; Dubois et al., 2014) in the evaluation of patients with cognitive decline, including quantification of Aβ and p-tau in the cerebrospinal fluid (CSF) (Molinuevo et al., 2018) and positron emission tomography (PET) (Johnson et al., 2012). In individuals with MCI, the detection of positive AD biomarkers is relevant for future planning, identifying suitable patients for clinical trials, and establishing early interventions (Weimer and Sager, 2009). Nevertheless, currently available AD biomarkers are expensive, invasive, and not widely accessible, usually restricted to applied and research settings (Whelan et al., 2022; Thijssen et al., 2022).

As a result, the evaluation of cognitive functions through neuropsychological tests has been extensively utilized as an accessible alternative to disease biomarkers for identifying individuals at high risk of developing AD (Espinosa et al., 2013; Alegret et al., 2013). Most studies have focused on evaluating memory and executive functions (Small et al., 1999; Buckner, 2004) as those are the cognitive deficits most affected during the disease continuum (Albert et al., 2011). Nevertheless, language alterations have also been shown as a sensitive hallmark of early cognitive impairment in AD (Taler and Phillips, 2008). For example, (Eyigoz et al., 2020) identified several linguistic parameters obtained in naturalistic probes as good prognostic markers for MCI. In Wang et al. (2022), the authors observed that the percentage of silenced pauses in the speech differed significantly across the different AD stages. Similarly, Mazzeo et al. (2022) showed that a single-word comprehension impairment could be an indicator for identifying patients who may need assistance with self-care in the upcoming years. Moreover, employing neuroimaging techniques, numerous studies have revealed alterations in brain connectivity (Montembeault et al., 2019; Rafiq et al., 2022; Wang et al., 2022) and atrophy (Smits et al., 2014; Wei et al., 2018) directly associated with language functions.

These findings, coupled with the digitalization experienced over the last few years, have increased the popularity of spontaneous speech (SS) protocols administered using digital tools (Beltrami et al., 2018; de la Fuente Garcia et al., 2019; Thomas et al., 2020). Classical cognitive assessments, based on traditional settings (i.e., neuropsychological batteries) usually demand the physical presence of clinicians in specialized health centers and are not always optimal for decentralized remote clinical trials (Tröger et al., 2022). In contrast, digital cognitive assessments are better-suited protocols when automated procedures are recommended or needed (Lindsay et al., 2021).

Among the numerous parameters that can be obtained computationally when applying a SS protocol, acoustic parameters (e.g., those derived from the speech waveform) are some of the most interesting in cognitive research. Patients with AD dementia (ADD) exhibit longer and more frequent hesitations, lower speech, and articulation rates, and longer pauses in SS tasks than non-demented individuals (Mueller et al., 2018). To integrate all the information extracted from SS, several approaches based on machine learning (ML) techniques have been applied. Tóth et al. (2018) adjusted models using SS in a recall task and found significant differences in speech tempo, articulation rate, silent pause, and length of utterance between early-stage ADD patients and healthy control individuals. Fraser et al. (2016) identified several voice abnormalities in speech related to ADD. Vocal and temporal features also demonstrated good discriminant properties when differencing among MCI, mild ADD, and moderate ADD (accuracy > 80%) (König et al., 2015). In a study using data from over 8700 participants, acoustic parameters generated from a simple reading task were found to differ among cognitively healthy individuals, MCI patients, and participants with global cognitive impairment, especially those with the lowest and higher degree of impairment (Nagumo et al., 2020).

Until now, most studies involving SS have prioritized the development of diagnostic tools, with a primary focus on identifying individuals with ADD (Asgari et al., 2017; Xue et al., 2021; Mahajan and Baths, 2021; He et al., 2023). Only a limited number of investigations have examined the application of SS in subjects with MCI, and even fewer studies have explored its association with biomarkers of interest, such as amyloid accumulation in the brain or CSF (Verfaillie et al., 2019; Mueller et al., 2021; Hajjar et al., 2023). In this context, Verfaillie et al. (2019) identified an association between high amyloid burden and fewer specific words during SS in 63 individuals with subjective cognitive decline (SCD) from a memory clinic. Mueller et al. (2021) showed that a positive amyloid status was longitudinally associated with poor achievement in several SS parameters (i.e., unique/total word production) using the Cookie Theft picture in a cohort of cognitively unimpaired individuals. Recently, Hajjar et al. (2023) explored the association between variables extracted from SS and amyloid status assessed by CSF in a population of cognitively healthy individuals and MCI using ML techniques. For the first time, the authors demonstrated that the SS can predict the amyloid status outperforming neuropsychological tests typically used to evaluate language obtaining an AUC of 0.77.

The present study aims to provide further evidence to address the existing gap between SS and the amyloid status quantified by CSF in an applied setting, using a sample of patients with MCI evaluated in a memory clinic. In particular, the Cookie Theft picture from the Boston Diagnostic Aphasia Examination was used as a speech task, ensuring easier standardization and maintaining great simplicity by analyzing only acoustic parameters (i.e., excluding lexico-syntatic parameters that are slower to analyze, more expensive, and frequently conditioned by more prior validation processes).

## 2. Materials and methods

The study had a cross-sectional design and included 52 patients with MCI who underwent clinical and neuropsychological evaluations, a lumbar puncture (LP) for the assessment of AD-core biomarkers in CSF, and lastly, a SS test using the acceXible platform.

### 2.1. Study participants

This study included 52 patients with a diagnosis of MCI (Petersen, 2004) who were evaluated at the memory clinic from Ace Alzheimer Center Barcelona (single site) between April 2022 and January 2023. Participants were either referred to the memory clinic by their general health practitioner due to cognitive problems (or subjective complaints) or they attended the open house initiative without the need for a physician's referral (Boada et al., 2014). All clinical and biomarker measures were obtained within a 6-month window from the SS protocol administration. This project is part of a study focused on the identification of risk factors of dementia through speech analysis (Tartaglia: MIA.2021.M02.0005).

### 2.2. Clinical assessment

Study participants completed neurological, neuropsychological, and social evaluations at the Ace Alzheimer Center Barcelona Memory Clinic and were followed up annually. A consensus diagnosis was assigned to each patient by a multidisciplinary team of professionals (Boada et al., 2014). Demographic information collected included age, sex, and years of formal education. The cognitive assessment included the Spanish version of the Mini-Mental State Examination (MMSE) (Folstein, 1992), the memory part of the Spanish version of the Seven Minute test (Del Ser et al., 2006), the Spanish version of the Neuropsychiatric Inventory Questionnaire (NPI-Q) (Boada et al., 2005), the Hachinski's ischemic score scale (Hachinski et al., 1974), the Blessed Dementia Scale (Blessed et al., 1968), and the Clinical Dementia Rating (CDR) scale (Morris, 1993), as well as the comprehensive Neuropsychological Battery of Fundació ACE (NBACE) (Alegret et al., 2012). MMSE (Folstein, 1992), and NBACE (Alegret et al., 2012) were assessed on all visits. At the baseline, all participants had a CDR of 0.5.

### 2.3. Neuropsychological assessment

Cognitive data were collected at the baseline using the NBACE. NBACE is a 45-min battery designed to assess cognitive domains especially affected in the elderly when cognitive impairment is suspected (Alegret et al., 2012). The NBACE was proposed as a brief, easy-to-administer and goal-directed compilation of globally-used neuropsychological tests in our target population. In this study, the following cognitive domains were explored: attention, information processing speed, verbal learning and memory, language, visuoperception, visuospatial ability, praxis, and executive functions. Normative data and cut-off scores of the NBACE subtests for individuals over 44 years old can be found elsewhere (Alegret et al., 2012, 2013).

### 2.4. Lumbar puncture and quantification of CSF core biomarkers for AD

Lumbar punctures (LPs) were performed at Ace Alzheimer Center Barcelona by an experienced neurologist under fasting conditions. The collection protocol follows the recommendations of the Alzheimers Biomarkers Standardization (Vanderstichele et al., 2012). The CSF was collected passively in 10 mL polypropylene tubes (Sarstedt Ref 62.610.018) and centrifuged (2000× g 10 min at 4 °C) within 2 h of acquisition. After centrifugation, the fluid was aliquoted into polypropylene tubes (Sarstedt Ref 72.694.007) and stored at -80 °C until analysis. The day of the analysis, one aliquot of 0.5 mL was thawed and used for the determination of Aβ1-42. Aβ1-42 protein was quantified by the commercially available chemiluminescense enzyme immunoassay (CLEIA) using the Lumipulse G 600 II automatic platform (Fujirebio Europe, Göteborg, Sweden) (Leit ao et al., 2019). Cutoffs from the Ace Alzheimer Center Barcelona CSF program were used to dichotomize Aβ1-42. A patient was considered amyloid positive when Aβ1-42 levels were <796 pg/mL (Orellana et al., 2022).

### 2.5. Recording protocol and preparation of voice data

Each participant performed the speaking task with the supervision of a neuropsychologist and using the acceXible platform app on a tablet. This app identifies vocal biomarkers for disease detection and monitoring. The image of the Cookie Theft picture was presented on the screen, and participants were asked to describe the image in detail. The voice was automatically recorded as part of an ongoing research protocol. The evaluations were conducted in Spanish and in a quiet environment. Participants audios were standardized to a frequency of 16KHz. Subsequently, the initial and final silences were automatically removed, and the deep learning model presented in Defossez et al. (2020) was applied to remove environmental noise. Acoustic features from the extended Geneva Minimalistic Acoustic Parameter Set (eGeMAPS) (v02) were extracted from every record using the open-source toolkit OpenSmile (v2.4.2) (Eyben et al., 2015). The set of features from the eGeMAPS are oriented to provide a simplified and standardized selection of relevant acoustic parameters for detecting physiological changes in voice production guided by findings of previous related studies (Scherer, 1986; Banse and Scherer, 1996). Appendix 1 includes the list of these 88 features.

### 2.6. Ethical considerations

This study and its informed consent were approved by the ethics committees of the Hospital Universitari de Bellvitge (Barcelona) (ref. PR007/22) under Spanish biomedical laws (Law 14/2007, 3 July, regarding biomedical research; Royal Decree 1716/2011, 18 November) and followed the recommendations of the Declaration of Helsinki. All participants signed an informed consent for the spontaneous speech protocol and for the lumbar puncture procedure.

The informed consent for the LP provides patients with information about the procedure, the most frequent side effects, and the primary objective of obtaining the AD-core biomarkers in CSF (which extends beyond clinical purposes and includes research interests). Twenty four h later after the LP, a member of the ACEs nursing team contacted the patients via phone to monitor any potential side effects and offer medical advice if needed. To ensure transparency, patients are also informed about the utilization of audio files collected during the speech test, including the primary research objectives and the security measures implemented for processing and storage on our servers or by our collaborators.

### 2.7. Data modeling

Statistical analyses were performed on STATA 15 (Stata Corporation, College Station, TX, USA) and ML modeling using Python (version 3.9.16).

Demographic, clinical, neuropsychological, and acoustic variables were contrasted between participants with positive and negative amyloid status using *t-test* or χ^2^ analyses. Logistic regression analyses were performed to evaluate the association of neuropsychological tests and acoustic variables with positive amyloid status. As all these bivariate and multivariate analyses were performed only for a descriptive purpose, no corrections for multiple testing were applied.

ML techniques were used for the prediction of amyloid-positive status using acoustic, neuropsychological, and demographic variables. The demographic variables considered were sex, age, and years of formal education. On the other hand, the neuropsychological variables included were total scores on similarities, digit forward, and digit backwards from the Wechsler Adult Intelligence Scale, third edition (WAIS-III) (Wechsler, 2002); long-term and recognition memory on the word list subtest from the Wechsler Memory Scale, third version (WMS-III) (Wechsler, 1997); the 15-Objects Test (Pillon et al., 1989); Poppelreuter-type overlap figures (Sala et al., 1995); the Automatic Inhibition Subtest of the Syndrom Kurtz Test (SKT) (time in s) (Erzigkeit, 1989); phonetic and semantic verbal fluencies (Artiola et al., 1999; Goodglass and Kaplan, 1972); an abbreviated 15-item naming test from the Boston Naming Test (BNT) (Kaplan et al., 2001); Verbal Comprehension (Alegret et al., 2012); and the Luria's Clock test (Golden, 1980) (variables were listed in Table 1). As input data for the models, three different sets of features were considered. The first dataset was based on the neuropsychological and demographic variables, the second included the eGeMAPS acoustic parameters (88 variables), and the third combined demographic and acoustic variables (92 variables). The aim of the first feature set, based on neuropsychological and demographic variables, was to establish a baseline model for comparing the capacity of the acoustic parameters for predicting the amyloid status.

**Table 1:** Demographic and clinical characteristics of the study participants.

**Variable**	**Mean (SD) or %**
Age	73.8 (8.5)
Sex (% females)	65.4
Years of formal education	8.8 (4.6)
MMSE score	27.0 (2.6)
(+) Amyloid status (%)	57.7
APOE ϵ4 carriers (%)	50.0
Amnestic MCI (%)	78.9
WAIS-III digit total forward	6.7 (1.5)
WAIS-III digit total backward	4.0 (1.4)
WMS-III delayed recall	2.1 (2.2)
WMS-III recognition task (total score)	19.5 (3.4)
The 15-objects test (correct answers)	10.3 (3.0)
Two Poppelreuter-type overlap figures (correct answers)	9.3 (1.1)
SKT (time in seconds)	37.0 (15.2)
Phonetic verbal fluency	10.5 (4.7)
Semantic verbal fluency	12.8 (5.0)
WAIS-III similarities	9.1 (3.3)
15-BNT free-evoked correct answers	13.1 (2.3)
Verbal comprehension	5.8 (0.5)
Luria's clock test (correct answers)	3.1 (1.1)

MMSE, Mini-Mental State Examination; WAIS-III, Wechsler Adult Intelligence Scale, Third Edition; WMS-III, Wechsler Memory Scale, third version; SKT, Syndrom Kurztest Test; BNT, Boston Naming Test.

For the datasets including acoustic variables, the following models were considered: (1) models with no previous feature engineering: Elastic Net (EN) and Random Forest (RF), (2) models combined with a prior dimensionality reduction using principal component analysis (PCA): EN, logistic regression (LR), support vector machines (SVM), and K-nearest neighbors (KNN), and (3) wrapper-based feature selection combining variable-length particle swarm optimization (VLPSO) (Tran et al., 2018) and KNN. Given the small number of input features in the dataset based on neuropsychological and demographic variables, the VLPSO feature selection strategy was not applied.

Briefly, the VLPSO is a wrapped-based feature selection algorithm (Xue et al., 2015). This population-based metaheuristic is used to remove irrelevant and redundant features in order to maximize the performance on a given task. In this study, the VLPSO algorithm was used to select variables maximizing the classification performance. A more detailed explanation of the VLPSO implementation used in this research can be found in Appendix 3. Moreover, Appendix 4 contains the hyperparameters of the algorithms used in this study.

**Figure 1:**
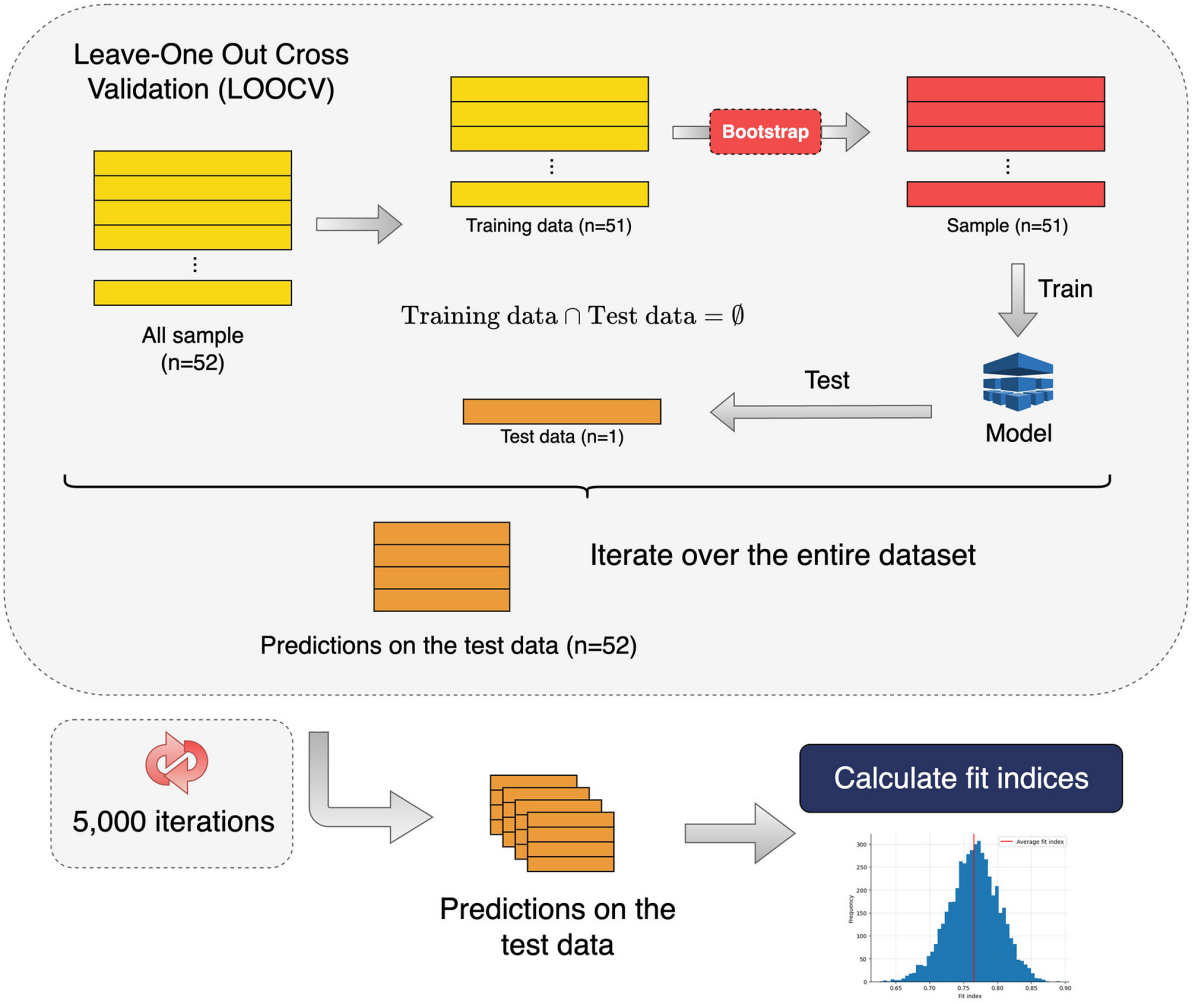
Pipeline used to evaluate the goodness-of-fit of all the models used to predict amyloid status. Performance metrics and confidence intervals were calculated from the metric distribution obtained after 5,000 iterations of a nested leave-one-out cross validation (LOOCV) where the training set used for adjusting the models was generated by bootstrapping.

To obtain a more reliable estimate of the goodness of fit of the models, they were evaluated by applying bootstrapping (5,000 iterations) to the training set with the leave-one-out cross validation (LOOCV) as shown in Figure 1. The Scikit-Learn (Buitinck et al., 2013) implementation was used for all the models described, except the VLPSO algorithm. The VLPSO code is available on GitHub.

## 3. Results

### 3.1. Statistical analysis

Demographic and clinical data of the 52 participants are described in Table 1. The cohort had a mean age of 73 years, 65% were female, and they completed a mean of 8.8 years of formal education. Thirty cases (57.7%) showed a positive amyloid status. Within those with a negative amyloid status, fifteen cases (68.2%) had a normal CSF profile and seven (31.8%) showed elevated t-tau and/or p-tau levels (suspected non-Alzheimer changes). Fifty percent of the sample were APOE ϵ4 carriers. Among subjects with a positive amyloid status, 56.6% carried an APOE ϵ4 allele, while for those with a negative status, the percentage decreased to 18.1%. The audio recording of the picture presentation had an average duration of 46 s (SD=15.6).

The bivariate contrasts for demographic and neuropsychological variables between participants with positive and negative amyloid status are shown in Table 2. Those participants with a positive amyloid status were significantly older, had fewer years of formal education, and when contrasting neuropsychological tests, they showed lower scores in the WMS-III, the 15-Objects Test, semantic verbal fluency, WAIS-III, BNT, and higher execution time of the SKT (all significant comparisons *p* < 0.043). To assess whether neuropsychological tests showed significant differences according to amyloid status controlling for the demographic characteristics of the sample, multivariate logistic models were applied adjusting the effect of neuropsychological tests by age, sex, and years of formal education. Amyloid status (positive/negative) was considered as the dependent variable. None of the neuropsychological variables maintained a significant effect in discriminating the amyloid status (Supplementary Table A2).

**Table 2:** Mean comparison of clinical and sociodemographic variables stratified by amyloid status.

**Variable**	**(+) Amyloid status Mean (SD) or %**	**(−) Amyloid status Mean (SD) or %**	**Statistic**	***p*-value**
Age	76.8 (4.5)	69.4 (10.6)	3.41	< .001^*^
Sex (% females)^*a*^	63.3	68.2	0.00	0.945
APOE ϵ4 carriers (%)^*a*^	56.6	18.1	6.29	0.012^*^
Years of formal education	7.4 (4.3)	10.6 (4.5)	2.56	0.013^*^
MMSE score	26.5 (2.3)	27.7 (2.8)	1.74	0.087
WAIS-III digit total forward	6.4 (0.9)	7.0 (1.9)	1.40	0.167
WAIS-III digit total backward	3.7 (1.1)	4.4 (1.6)	1.80	0.076
WMS-III delayed recall	1.2 (1.7)	3.2 (2.2)	3.64	< 0.001^*^
WMS-III recognition task (total score)	18.4 (3.6)	21.0 (2.4)	2.96	0.004^*^
The 15-objects test (correct answers)	9.4 (3.1)	11.3 (2.4)	2.34	0.023^*^
Two Poppelreuter-type overlap figures (correct answers)	9.1 (1.1)	9.5 (0.8)	1.52	0.132
SKT (seconds)	40.7 (16.2)	32.0 (12.2)	2.09	0.040^*^
Phonetic verbal fluency	9.4 (5.0)	11.9 (3.8)	1.95	0.055
Semantic verbal fluency	11.6 (4.8)	14.4 (4.8)	2.07	0.042^*^
WAIS-III similarities	7.8 (2.8)	10.8 (3.0)	3.70	< 0.001^*^
15-BNT free-evoked correct answers	12.3 (2.5)	14.1 (1.2)	3.08	0.003^*^
Verbal comprehension	5.7 (0.5)	5.8 (0.3)	0.87	0.385
Luria's clock test (correct answers)	3.0 (1.1)	3.2 (1.1)	0.70	0.483

Mean comparisons of quantitative variables were performed using a two-sample *t-test*, categorical variables were compared by a χ^2^ test^*a*^. MMSE, Mini-Mental State Examination; WAIS-III, Wechsler Adult Intelligence Scale, Third Edition; WMS-III, Wechsler Memory Scale, third version; SKT, Syndrom Kurztest Test; BNT, Boston Naming Test. ^*^Statistically significant (*p*-value < 0.05).

The same bivariate contrasts for acoustic features between participants with positive and negative amyloid status are depicted in Supplementary Table A1. Five of the 88 acoustic features analyzed showed a significant difference between the two groups. These variables included the F3-bandwidth (voiced - coefficient of variation), the Hammarberg index (unvoiced - mean), the Alpharatio (unvoiced - mean), and the Voiced-Segment-Length/second (mean and std) (Eyben et al., 2015). When adjusting these variables for age, sex, and years of education, only F3-bandwidth (voiced - coefficient of variation) became non-significant (*p* > 0.05) (Supplementary Table A3).

### 3.2. Machine learning analysis

The fit indices of the two best models obtained for differentiating the amyloid status across the three different datasets (neuropsychological and demographic / acoustic / acoustic and demographic) by applying the evaluation strategy depicted in Figure 1 are reported in Table 3. The area under the curve (AUC) was used as the reference metric. The best AUC value was observed for the model that included only acoustic variables, followed by the model based on acoustic and demographic parameters. The models with the best performance were those incorporating the wrapper-based feature selection strategy (VLPSO+KNN) reaching AUCs of 0.79 (_95_CI: [0.71-0.86]) (acoustic) and 0.74 (_95_CI: [0.66-0.82]) (acoustic and demographic). These two models evaluated by simply performing a LOOCV without bootstrapping achieved AUCs of 0.83 and 0.79, respectively. In contrast, models based on demographic and neuropsychological variables performed poorly, with AUCs below 0.7 and accuracies close to 60%.

**Table 3:** Fit indices of the two best models obtained for each of the explored feature sets.

**Feature set**	**Model**	**Accuracy**	**Precision**	**Recall**	**F1^*a*^**	**AUC**
Neuropsychological and demographic	PCA(9) + EN	0.63 [0.54–0.72]	0.67 [0.60–0.75]	0.71 [0.59–0.83]	0.69 [0.60–0.77]	0.66 [0.58–0.74]
	PCA(9) + KNN	0.63 [0.52–0.73]	0.66 [0.58–0.74]	0.72 [0.58–0.87]	0.69 [0.59–0.79]	0.62 [0.51-0.72]
Acoustic	VLPSO + KNN	0.75 [0.67–0.83]	0.76 [0.69–0.83]	0.82 [0.71–0.93]	0.79 [0.72–0.86]	0.79 [0.71–0.86]
	RF	0.64 [0.55–0.74]	0.68 [0.59–0.76]	0.73 [0.61–0.85]	0.70 [0.62–0.79]	0.64 [0.55–0.74]
Acoustic and demographic	VLPSO + KNN	0.72 [0.63–0.81]	0.74 [0.66–0.81]	0.81 [0.70–0.92]	0.77 [0.69–0.85]	0.74 [0.66–0.82]
	RF	0.65 [0.55–0.75]	0.68 [0.60–0.77]	0.74 [0.62–0.86]	0.71 [0.62–0.80]	0.65 [0.56–0.75]

Metrics and confidence intervals were calculated using 5,000 bootstrap iterations with a nested LOOCV. AUC, area under the curve; EN, ElasticNet; PCA(C), principal component analysis using the first C components; VLPSO, variable-length particle swarm optimization; KNN, K-nearest neighbors; RF, random forest. Model hyperparameters are provided in Appendix 4.

${}^{a}\text{F1-score}=2\cdot \frac{\text{Precision}\cdot \text{Recall}}{\text{Precision}+\text{Recall}}$.

**Figure 2:**
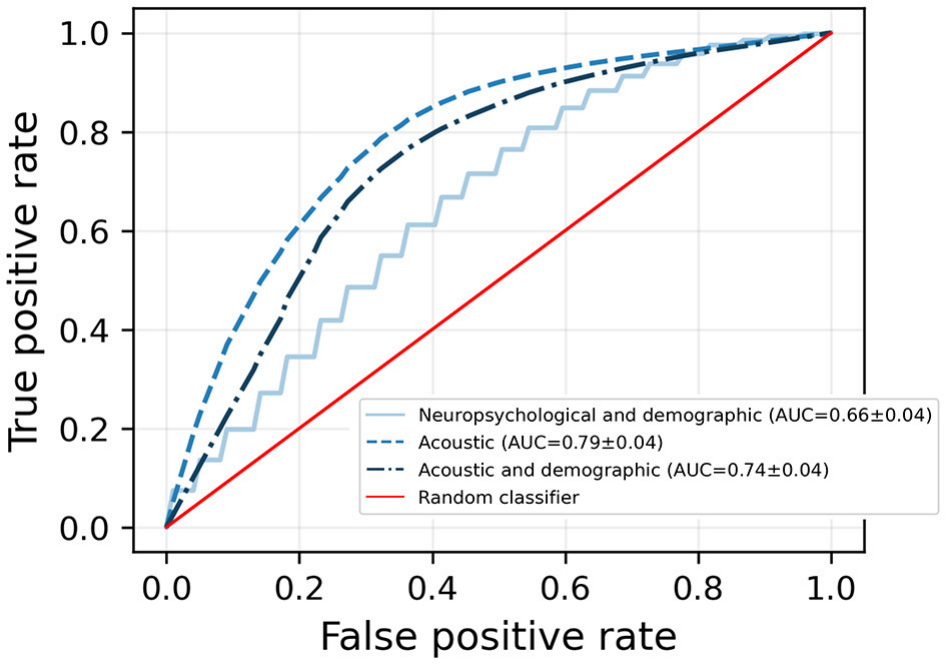
Receiver operating characteristic (ROC) curve for predicting amyloid status based on demographic and neuropsychological, acoustic, and a combination of demographic and acoustic variables. The results correspond to the best models presented in Table 3. For each model, the mean AUC calculated by 5,000 bootstrap iterations as described in Figure 1 is shown. AUC: area under the curve.

**Figure 3:**
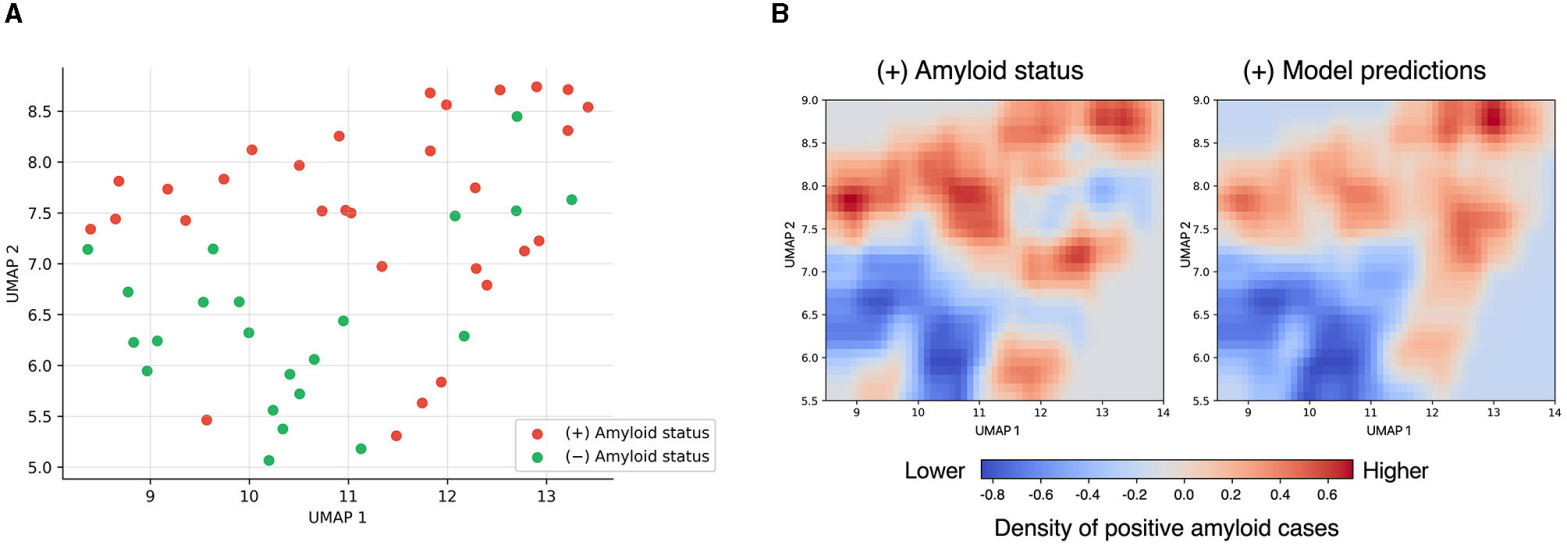
Uniform manifold approximation and projection (UMAP) (Sainburg et al., 2020) dimensionality reduction of the most discriminative feature set obtained by the VLPSO feature selection algorithm. **(A)** Projection highlighting positive and negative amyloid status. **(B)** Projection of the K-nearest neighbor decision boundary. On the left is the density plot representing the higher presence (red) or absence (blue) of amyloid-positive cases in the data. On the right are shown the KNN predictions of amyloid-positivity, where red indicates that the model assigns a higher probability of amyloid-positivity and blue a lower probability. UMAP hyperparameters: number of neighbors = 8 and minimum distance = 0.1; the rest of the hyperparameters were left as default.

The dimensionality reduction achieved by the VLPSO-based models went from 88 features to 7 and 10 for the datasets based on acoustic features and combining acoustic and demographic information, respectively. Figure 2 shows the receiver operating characteristic (ROC) curves of the best models obtained for each dataset (see Table 3). The two-dimensional projection of the features associated with the best model from Table 3 (VLPSO+KNN using acoustic variables) and their decision boundary is illustrated in Figure 3.

**Figure 4:**
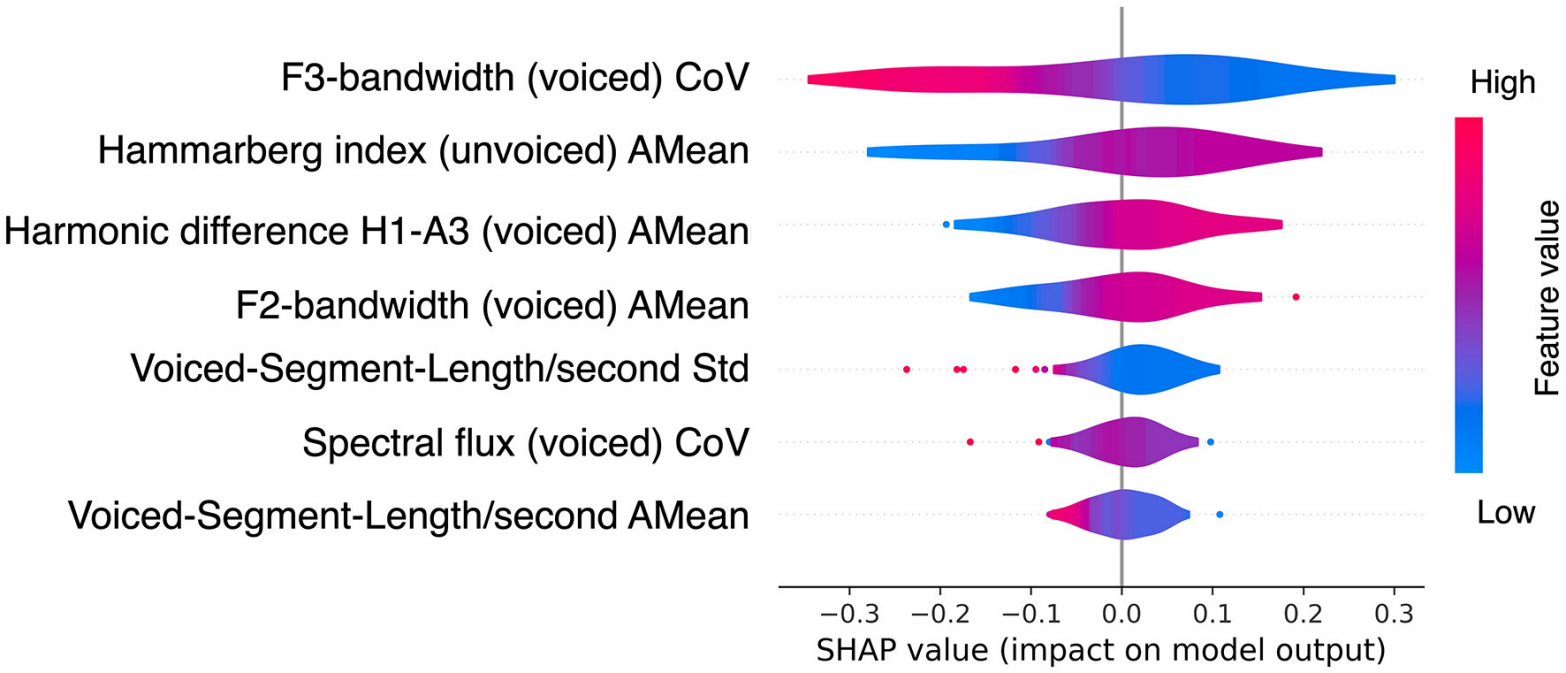
SHapley Additive exPlanations (SHAP) values Lundberg and Lee (2017) of the best model for predicting amyloid status. The SHAP values were calculated on the test set using LOOCV. The best model corresponds to the combination of VLPSO with KNN using acoustic variables (see Table 3). The feature color indicates how it relates to the probability of being amyloid positive or negative. The red color is associated with higher feature values, while blue is associated with lower values. Thus, lower values in the F3-bandwidth (voiced) CoV (blue area) are associated with an increased likelihood of being amyloid positive; and having a lower mean of the spectral Hammarberg index (voiced) (red area) is associated with a lower probability of being amyloid positive. CoV: coefficient of variation; AMean: arithmetic mean; Std: standard deviation.

The most discriminant features for differentiating the amyloid status were further analyzed using SHapley Additive exPlanations (SHAP) (Lundberg and Lee, 2017). Figure 4 shows the impact of each variable on the amyloid status probability. Variables were ordered based on the average absolute SHAP value in a descending order. Positive SHAP values are associated with a higher probability of a positive amyloid status, while negative values with a lower probability. The most relevant features for predicting a positive status were the frequency based: F3-bandwidth (voiced–coefficient of variation) and F2 bandwidth (voiced–mean); the spectral-based: Hammarberg index (unvoiced–mean), harmonic difference H1-A3 (voiced–mean), and spectral flux (voiced–coefficient of variation); and the temporal-based: voiced-segment-length/second (mean and std).

## 4. Discussion

This study investigated the association between the amyloid status assessed by CSF and voice features derived from the use of the Cookie Theft picture description in a cohort of 52 MCI patients evaluated in a memory clinic. We found noticeable differences in physicoacoustic characteristics between patients with positive and negative amyloid status using a widely extended SS test. The analysis unveiled statistical variations in multiple acoustic parameters, and the applied ML models showed a good discriminatory capacity for predicting amyloid positivity (Table 3). Furthermore, by incorporating XAI techniques, we gained valuable insights into how different input variables influenced the decisions made by the models (Figure 4).

As shown in the UMAP projection (Figure 3), the features identified by our ML-based feature selection algorithm provided a clear distinction of amyloid status, leading to a good classification performance using a simple distance-based algorithm such as KNN. Interestingly, including sociodemographic variables did not improve the discriminatory capacity of the models, supporting that SS-derived information alone can act as a good predictor of amyloid status (AUC of 0.74 [0.66–0.82] vs. AUC 0.79 [0.71–0.86]). Moreover, we showed that SS features outperformed the conventional neuropsychological tests typically used to evaluate cognitive functions (Figure 2). These results provide evidence that the differences between positive and negative amyloid status in MCI subjects can be captured by aspects related to voice production.

Our results align with recent studies conducted by Hajjar et al. (2023) in a longitudinal cohort of cognitive unimpaired and MCI subjects, Mueller et al. (2018) in a longitudinal study involving healthy and early-stage MCI patients, and Verfaillie et al. (2019) in a cross-sectional study of individuals with cognitive decline. These studies demonstrated that amyloid burden is associated with several speech parameters. We also extended the relationship between the presence of brain amyloidosis, one of the main neuropathological hallmarks of AD, and speech parameters in MCI patients, providing a new landmark for the use of spontaneous speech in neurodegenerative disorders. Notably, the studies of Mueller et al. (2018) and Verfaillie et al. (2019) were based on the lexico-syntactic content of the speech, while our study focuses on the properties of the sound generated when describing a picture. The main idea here is that the analysis of speech and language could provide relevant information about the underlying pathophysiological process of AD (Voleti et al., 2019).

During the preclinical stage of AD, before symptom onset, the pathophysiological course of the disease is characterized by first the formation of amyloid plaques and later p-tau protein aggregates, which accumulate in the brain and disrupt normal neuronal function (Sperling et al., 2011). Subsequently, at the MCI stage, the accumulation of these proteins in the brain reaches a critical threshold, leading to neuronal injury and pathological changes in the volumes of different brain regions (Sperling et al., 2011). The most prominent cognitive deficits in MCI are typically in the domains of memory and executive function, which include abilities such as planning, decision-making, and problem-solving. However, it is reasonable to assume that more diverse and silent changes are taking place (Wilson and Petkov, 2011). In Mazzeo et al. (2022), researchers observed associations between disease progression, language lesions, and brain hypometabolism. In addition, evidence shows that MCI patients show longer speech and phonation time (Tóth et al., 2018; Gosztolya et al., 2019), an incremented length of silent pauses (voiceless) (Wang et al., 2022), lower speech rate (Tóth et al., 2018), presence of stammers and articulatory disfluencies that interrupt speech with longer hesitations (López-de Ipiña et al., 2013; Tóth et al., 2018), and impairments in formant features in phonological planning formants (Themistocleous et al., 2018). For a more detailed description of these alterations, (see Martínez-Nicolás et al., 2021). In our study, several voice parameters were identified as the most discriminatory using ML approaches for differentiating MCI with positive and negative amyloid status. Among the most important voice parameters identified (Figure 4), there were spectral features (relative energy in different frequency bands), associated with vocal emotional expre ssions Sauter et al. (2010), voiced segments (the portion of speech with relatively constant phonetic features) useful to differentiate AD from healthy individuals (López-de Ipiña et al., 2013; Wang et al., 2022), and measures of the Hammarberg index, a spectral measure of voice quality, which has been identified as a discriminant feature for MCI (Themistocleous et al., 2020).

The present study was unable to detect an association between amyloid status and conventional language tests included in the NBACE, such as BNT-15 or semantic verbal fluency. This result is concordant with those observed in MCI (Hajjar et al., 2023), cognitively healthy individuals (Baker et al., 2017) and those with subjective cognitive decline (Verfaillie et al., 2019). In fact, in our study, no neuropsychological test from NBACE showed a multivariate association with the amyloid status in MCI, and predictive models based on neuropsychological tests exhibited a lower discriminative capacity. These findings suggest that SS assessments may offer a more ecological and closely connected real-world representation of cognitive status for predicting Aβ42 status than traditional language evaluations, being of particular interest in the preclinical stages of AD. It is worth noting that our study exclusively focused on parameters that capture the structure and dynamics of the speech, without relying on syntactic and lexical information derived from voice recordings. This aspect is particularly significant as it enables the automated evaluation of information obtained from SS assessments, eliminating the requirement for manual language analysis.

As exposed before, the assessment of SS can be approached in different ways from narrower and more specific questions (i.e., “describe the presented image") to more open-ended elicitations (i.e., “describe the happiest moment of your life”). However, these open-ended approaches are subjected to more individual and contextual factors, resulting in more variability and limiting their generalizability when contrasting results between studies (Mueller et al., 2018). More efforts should be devoted to providing standards and protocols to improve the accuracy of procedures and algorithms and to stimulate the integration of innovative solutions in SS processing to clinical practice or trials (Haider et al., 2019; Tröger et al., 2022). In this sense, it should be noted that one of the strengths of the present study was to provide precise discriminant results by distinguishing the amyloid status (positive vs negative) in a relevant clinical population (patients with MCI) in an applied setting (a memory unit), using a simple speech strategy (description of a picture), through a very well-known and accessible tool (the Cookie Theft picture), administered during less than 1 min, and focusing attention only on acoustic features, obtained using a set of standardized variables (eGeMAPS) (Eyben et al., 2015).

We acknowledge that our study has certain limitations. First, the small sample size restricts the generalization of these results and should be treated with caution. Although the models were evaluated following an exhaustive bootstrap and cross-validation approach to obtain a more realistic approximation of their performance, larger sample sizes are required to confirm our results. Additionally, our findings were based on cross-sectional data, while the relationship between CSF biomarkers and SS is probably complex and multifactorial. Further research is required to understand the longitudinal association between amyloid burden and voice (and other speech) features, investigating the evolution of language parameters using follow-up information. Moreover, our study solely focused on predicting amyloid status in CSF, the primary pathological hallmark of AD and a key target for drug development (Alzheimer's & Dementia, 2023). However, extending the presented analytical framework to include other CSF (e.g., p-tau 181 or total tau) or neuroimaging biomarkers (e.g., hypometabolism, tau accumulation, or atrophy) is of great interest for future investigations (Scheltens et al., 2021). For example, it has been demonstrated that tau levels exhibit a stronger correlation with cognitive alterations compared to amyloid levels (Aschenbrenner et al., 2018). In addition, prior studies have shown that reductions in brain metabolism (Vanhoutte et al., 2017) or changes in activation patterns measured by fMRI (Vanhoutte et al., 2017) occur in the early stages of the disease and are associated with language production. Therefore, while the primary aim of this study was to provide an initial approximation of the predictive capacity of amyloid status from SS in MCI patients, the development of future predictive models should consider a broader panel of disease biomarkers. Finally, using the Cookie Theft picture description facilitated standardized results. Nevertheless, relying on a unique test imposes limitations when characterizing language impairment. Consequently, incorporating additional SS tests within the models should improve their predictive performance.

Despite these limitations and in the context of accumulated data, such as that provided in the present study, it is possible to foresee promising horizons for the application of voice processing technology. Based on SS and IA techniques, the identification of individuals at high risk of developing AD dementia could be accessible to clinicians by the longitudinal analysis of conversations. For example, specific speech tasks could be periodically administered remotely when other cognitive assessments are not feasible or when biomarker-based evaluations are either too expensive, non-accessible, or unsuitable for a particular patient. These advancements have the potential to make the identification of high-risk individuals more accessible to clinicians and significantly contribute to public health.

## 5. Conclusion

In conclusion, acoustic features derived from the Cookie Theft picture description are consistently associated with amyloid status assessed by CSF in MCI patients in the setting of a memory clinic. These results offer a new window of opportunities, focused on identifying, in a widely accessible, rapid, and non-invasive manner, the underlying biochemical status in patients with MCI providing information about their future cognitive progression and risk of conversion to dementia. Such advancements in early detection and monitoring of MCI can significantly impact clinical practice, enabling timely interventions and personalized treatment strategies. Further research is needed to validate and refine the SS protocols and explore their utility in larger and more diverse populations. Ultimately, this technology has the potential to bring us closer to improved diagnostic and prognostic tools for individuals with MCI.

## Data availability statement

The raw data supporting the conclusions of this article will be made available by the authors, without undue reservation.

## Ethics statement

The study involving humans was approved by Hospital Universitari de Bellvitge (PR007/22). The study was conducted in accordance with the local legislation and institutional requirements. The participants provided their written informed consent to participate in this study.

## Author contributions

SV, FG-G, MM, LT, AR, and MB: conceptualization. SV and FG-G: methodology and formal analysis. SV, FG-G, AC, IR, PG-G, CO, RP, AO, LM, and AG-S: data curation. SV, FG-G, MM, NM, MA, CZ, PG, WH, and AR: writing—review and editing. MM, NM, MA, VP, MR, CZ, PG, and NL: project administration. SV: supervision. All authors contributed to interpretation of the findings, the critical review of the manuscript, and have read and agreed to the published version of the manuscript.
