# Supplementary material for: Harnessing acoustic speech parameters to decipher amyloid status in individuals with mild cognitive impairment
Source: Front Neurosci. 2023 Sep 7;17:1221401. doi: 10.3389/fnins.2023.1221401 (PMC10512723; doi:10.3389/fnins.2023.1221401)
Supplement: Supplementary file 1 [file Data_Sheet_1.PDF]

Appendix 1

Bivariate contrasts between subjects with positive (N=22) and negative (N=30) amyloid status for the different acoustic variables extracted from the extended Geneva Minimalistic Acoustic Parameter Set (eGeMAPs) [1] are reported. A t-test was used to compare population means.

Table A1: Mean comparison of acoustic variables stratified by amyloid status.

Parameter Group	Variable	(+) Amyloid status Mean (SD)	(−) Amyloid status Mean (SD)	Statistic	p-value
Cepstral	MFCC1 (voiced) AMean	38.9 (2.6)	37.4 (3.3)	1.79	0.078
	MFCC1 (voiced) CoV	0.2 (0.0)	0.2 (0.0)	1.33	0.188
	MFCC1 AMean	22.8 (5.3)	21.4 (5.9)	0.87	0.387
	MFCC1 CoV	0.8 (0.2)	0.9 (0.2)	0.73	0.466
	MFCC2 (voiced) AMean	6.3 (5.1)	5.9 (7.0)	0.23	0.812
	MFCC2 (voiced) CoV	2.3 (2.1)	1.4 (2.5)	1.46	0.149
	MFCC2 AMean	4.0 (3.4)	3.9 (3.8)	0.08	0.931
	MFCC2 CoV	3.3 (2.6)	2.8 (2.9)	0.62	0.532
	MFCC3 (voiced) AMean	16.0 (6.3)	16.5 (5.5)	0.30	0.763
	MFCC3 (voiced) CoV	1.1 (0.4)	1.0 (0.3)	0.45	0.650
	MFCC3 AMean	11.0 (4.6)	10.7 (3.8)	0.23	0.813
	MFCC3 CoV	1.3 (0.5)	1.3 (0.3)	0.05	0.959
	MFCC4 (voiced) AMean	3.3 (7.7)	2.6 (10.0)	0.25	0.797
	MFCC4 (voiced) CoV	-0.1 (4.7)	-1.3 (3.8)	1.00	0.319
	MFCC4 AMean	3.6 (4.6)	3.3 (6.4)	0.19	0.843
	MFCC4 CoV	1.1 (4.3)	1.7 (5.1)	0.49	0.619
Energy/Amplitude	HNR (voiced) AMean	7.5 (2.1)	7.8 (1.4)	0.39	0.691
	HNR (voiced) CoV	0.3 (0.0)	0.3 (0.0)	0.72	0.470
	Loudness 20-80th percentile range	0.9 (0.2)	1.0 (0.2)	0.50	0.618
	Loudness 20th percentile	0.0 (0.0)	0.0 (0.0)	0.60	0.548
	Loudness 50th percentile	0.3 (0.2)	0.3 (0.2)	0.08	0.934
	Loudness 80th percentile	1.0 (0.2)	1.0 (0.2)	0.66	0.510
	Loudness AMean	0.5 (0.1)	0.5 (0.1)	0.35	0.721
	Loudness CoV	1.0 (0.2)	1.1 (0.3)	0.35	0.725
	Loudness Mean falling slope	8.8 (2.0)	8.8 (1.9)	0.00	0.996
	Loudness Mean rising slope	11.1 (2.4)	11.8 (2.9)	0.93	0.355
	Loudness Std falling slope	5.9 (1.0)	6.0 (1.3)	0.43	0.663
	Loudness Std rising slope	6.9 (1.5)	7.2 (1.7)	0.63	0.528
	Shimmer-localdb (voiced) AMean	1.0 (0.1)	0.9 (0.1)	1.69	0.095
	Shimmer-localdb (voiced) CoV	0.9 (0.1)	0.9 (0.1)	0.62	0.533
Frequency	F0-semitonefrom-27.5hz (voiced) 20-80th percentile range	3.9 (1.0)	4.0 (1.2)	0.28	0.779
	F0-semitonefrom-27.5hz (voiced) 20th percentile	27.9 (4.7)	27.9 (3.5)	0.00	0.996
	F0-semitonefrom-27.5hz (voiced) 50th percentile	29.7 (4.8)	29.6 (3.6)	0.08	0.933
	F0-semitonefrom-27.5hz (voiced) 80th percentile	31.9 (5.0)	32.0 (3.7)	0.06	0.949
	F0-semitonefrom-27.5hz (voiced) AMean	29.9 (4.7)	30.1 (3.5)	0.12	0.897

Variables were calculated from the 18 Low Level Descriptors (LLD) described in [1] using a symmetric moving average filter of three frames long. Mean comparisons were performed using a two-sample t-test. MFCC: Mel Frequency Cepstral Coefficient; AMean: Arithmetic Mean; CoV: Coefficient of Variation; HNR: Harmonics-to-Noise Ratio.
*Statistically significant (p-value < 0.05).

Table A1: (continued)

Parameter Group	Variable	(+) Amyloid status Mean (SD)	(−) Amyloid status Mean (SD)	Statistic	p-value
Frequency	F0-semitonefrom-27.5hz (voiced) CoV	0.1 (0.0)	0.1 (0.0)	0.55	0.580
	F0-semitonefrom-27.5hz (voiced) Mean falling slope	55.4 (30.1)	55.3 (40.8)	0.00	0.993
	F0-semitonefrom-27.5hz (voiced) Mean rising slope	152.5 (93.9)	135.1 (77.6)	0.70	0.482
	F0-semitonefrom-27.5hz (voiced) Std falling slope	91.1 (70.3)	81.8 (75.0)	0.45	0.648
	F0-semitonefrom-27.5hz (voiced) Std rising slope	209.9 (138.8)	211.4 (155.1)	0.03	0.972
	F1-amplitudelogrelf0 (voiced) AMean	-116.5 (22.5)	-114.4 (23.1)	0.32	0.745
	F1-amplitudelogrelf0 (voiced) CoV	-0.8 (0.1)	-0.8 (0.1)	0.39	0.692
	F1-bandwidth (voiced) AMean	1382.4 (51.1)	1380.7 (35.9)	0.12	0.897
	F1-bandwidth (voiced) CoV	0.1 (0.0)	0.1 (0.0)	0.10	0.919
	F1-frequency (voiced) AMean	585.9 (62.6)	570.0 (47.2)	0.99	0.324
	F1-frequency (voiced) CoV	0.3 (0.0)	0.3 (0.0)	0.54	0.586
	F2-amplitudelogrelf0 (voiced) AMean	-120.5 (20.4)	-118.5 (21.4)	0.33	0.741
	F2-amplitudelogrelf0 (voiced) CoV	-0.7 (0.1)	-0.7 (0.1)	0.47	0.638
	F2-bandwidth (voiced) AMean	1088.6 (53.7)	1062.8 (49.9)	1.75	0.085
	F2-bandwidth (voiced) CoV	0.2 (0.0)	0.3 (0.0)	0.95	0.342
	F2-frequency (voiced) AMean	1666.3 (72.4)	1660.2 (59.3)	0.32	0.746
	F2-frequency (voiced) CoV	0.1 (0.0)	0.1 (0.0)	0.23	0.812
	F3-amplitudelogrelf0 (voiced) AMean	-123.9 (19.5)	-121.3 (20.7)	0.45	0.647
	F3-amplitudelogrelf0 (voiced) CoV	-0.6 (0.1)	-0.6 (0.1)	0.75	0.451
	F3-bandwidth (voiced) AMean	1043.3 (62.4)	1029.9 (61.0)	0.77	0.442
	F3-bandwidth (voiced) CoV	0.2 (0.0)	0.3 (0.0)	2.74	0.008*
	F3-frequency (voiced) AMean	2736.7 (97.9)	2725.1 (64.2)	0.48	0.628
	F3-frequency (voiced) CoV	0.0 (0.0)	0.0 (0.0)	0.45	0.652
	Jitter-local (voiced) AMean	0.0 (0.0)	0.0 (0.0)	1.64	0.106
	Jitter-local (voiced) CoV	1.5 (0.4)	1.5 (0.3)	0.29	0.771
Spectral	Alpharatio (voiced) AMean	-19.5 (± 2.6)	-18.4 (± 2.5)	1.52	0.132
	Alpharatio (voiced) CoV	-0.4 (0.0)	-0.4 (0.0)	1.35	0.180
	Alpharatio (unvoiced) AMean	-6.3 (2.1)	-4.6 (1.8)	3.07	0.003*
	Hammarberg index (voiced) AMean	29.8 (3.4)	28.3 (2.6)	1.75	0.085
	Hammarberg index (voiced) CoV	0.3 (0.0)	0.3 (0.0)	1.89	0.063
	Hammarberg index (unvoiced) AMean	16.6 (2.0)	14.3 (2.2)	3.86	0.000*
	Harmonic difference H1-A3 (voiced) AMean	29.9 (4.4)	28.0 (3.3)	1.71	0.092
	Harmonic difference H1-A3 (voiced) CoV	0.3 (0.0)	0.3 (0.0)	0.39	0.695
	Harmonic difference H1-H2 (voiced) AMean	5.4 (4.5)	4.8 (3.6)	0.57	0.570
	Harmonic difference H1-H2 (voiced) CoV	1.3 (1.2)	1.4 (1.3)	0.07	0.940
	Slope0-500 (unvoiced) Amean	0.0 (0.0)	0.0 (0.0)	0.49	0.619
	Slope0-500 (voiced) AMean	0.0 (0.0)	0.0 (0.0)	0.24	0.804
	Slope0-500 (voiced) CoV	0.5 (0.5)	0.6 (0.5)	0.29	0.768
	Slope500-1500 (unvoiced) Amean	0.0 (0.0)	0.0 (0.0)	0.42	0.672
	Slope500-1500 (voiced) AMean	-0.0 (0.0)	-0.0 (0.0)	0.65	0.514
	Slope500-1500 (voiced) CoV	-0.7 (0.2)	-0.7 (0.2)	0.72	0.471
	Flux (unvoiced) Amean	0.1 (0.0)	0.1 (0.0)	1.30	0.198
	Flux (voiced) AMean	0.5 (0.0)	0.5 (0.1)	1.09	0.278
	Flux (voiced) CoV	0.6 (0.0)	0.6 (0.0)	1.50	0.138
	Flux AMean	0.3 (0.0)	0.2 (0.0)	0.91	0.364
	Flux CoV	1.2 (0.2)	1.3 (0.3)	0.62	0.531
Temporal	Temporal-feature Loudness-Peak/second	2.5 (0.7)	2.3 (0.7)	0.87	0.384
	Unvoiced-Segment-Length/second AMean	0.3 (0.1)	0.4 (0.2)	0.83	0.405
	Voiced-Segment-Length/second AMean	0.2 (0.0)	0.3 (0.0)	2.42	0.019*
	Unvoiced-Segment-Length/second Std	0.6 (0.4)	0.7 (0.5)	0.29	0.770
	Voiced-Segment-Length/second Std	0.2 (0.0)	0.2 (0.0)	2.95	0.004*
	Voiced-Segments/second	1.6 (0.4)	1.5 (0.5)	1.20	0.233
Others	Others Equivalent-Sound-Level (dB)	-19.1 (± 1.7)	-19.4 (± 1.8)	0.63	0.525

Variables were calculated from the 18 Low Level Descriptors (LLD) described in [1] using a symmetric moving average filter of three frames long. Mean comparisons were performed using a two-sample t-test. MFCC: Mel Frequency Cepstral Coefficient; AMean: Arithmetic Mean; CoV: Coefficient of Variation; HNR: Harmonics-to-Noise Ratio.

*Statistically significant (p-value < 0.05).

Appendix 2

This section includes the multivariate analyses performed to examine the effect of the neuropsychological tests and physical-acoustic variables that showed significant differences in the bivariate analyses shown in Tables A and B, respectively. Logistic regression models were used considering as the dependent variable the amyloid status (positive = 1) adjusting for age, sex, and years of formal education.

Table A2: Logistic regression model based on neuropsychological tests for predicting amyloid positive status.

Variable	Odss Ratio	$_{95\%}$CI	p-value
WMS-III delayed recall	0.886	[0.490, 1.602]	0.737
WMS-III recognition task (total score)	0.794	[0.617, 1.022]	0.134
The 15-Objects test (correct answers)	0.978	[0.716, 1.335]	0.905
SKT (seconds)	0.980	[0.922, 1.043]	0.598
Semantic verbal fluency	1.225	[0.980, 1.517]	0.120
WAIS-III Similarities	0.748	[0.510, 1.097]	0.213
15-BNT free evoked correct answers	0.448	[0.229, 0.878]	0.050

Statistically significant neuropsychological variables from Table 2 were considered for analysis. Variables were adjusted by age, sex, and years of formal education. WMS: Wechsler Memory Scale; SKT: Syndrom Kurztest Test; BNT: Boston Naming Test.

Table A3: Logistic regression model based on acoustic variables for predicting amyloid positive status.

Variable	Odss Ratio	$_{95\%}$CI	p-value
Frequency F3-bandwidth (voiced) CoV	0.641	[0.437, 0.939]	0.056
Spectral Hammarbergindex (unvoiced) AMean	1.702	[1.234, 2.347]	0.007
Temporal-feature Voiced-Segment-Length/second Mean	0.726	[0.582, 0.905]	0.017
Temporal-feature Voiced-Segment-Length/second Std	0.710	[0.554, 0.910]	0.023
Spectral Alpharatio (unvoiced) AMean	0.615	[0.445, 0.850]	0.014

Statistically significant acoustic variables from Table A1 were considered for analysis. Variables were adjusted by age, sex, and years of formal education.

Appendix 3

To reduce the number of features, a wrapper-based approach was adopted using a similar algorithm as the one presented in [2]. This appendix provides a brief context about feature selection and details the implementation used in this work.

Feature selection algorithms aim to find the best combination of features by removing irrelevant/redundant variables while preserving its original meaning. The feature selection process can be divided into two phases: subset generation (SG) and subset evaluation (SE) [3]. In this work, the SE part was accomplished through a K-Nearest Neighbor (KNN) algorithm[1] evaluated by Leave-One-Out Cross Validation (LOOCV). In this setting, the Area Under the Curve (AUC) was used as a performance metric. On the other hand, the SG part was performed using a Variable-Length Particle Swarm Optimization (VLPSO) algorithm [2].

This appendix is intended to provide the necessary information to replicate the implemented strategy. For more information about PSO algorithms or the original VLPSO approach, the reader is referred to [5, 6, 7] and [2]. All the code is available on GitHub.

PSO algorithms are population-based metaheuristic optimization strategies. These algorithms are based on a swarm of particles representing candidate solutions. At each iteration of the algorithm, the parameters associated with each particle are updated to optimize a given objective function. In the context of the feature selection performed in this work, the optimization aimed to maximize the AUC obtained during the SE phase.

Each particle in the swarm has several vectors with the following information: current position, $\boldsymbol{p}_{\text{curr}} \in \mathbb{R}^L$, velocity $\boldsymbol{v} \in \mathbb{R}^L$, best position so far $\boldsymbol{p}_{\text{best}} \in \mathbb{R}^L$, best swarm's position $\boldsymbol{g}_{\text{best}} \in \mathbb{R}^L$, and what the authors of [2] called *exemplar* $\boldsymbol{p}_{\text{expl}} \in \mathbb{R}^L$, being L the dimensionality of the particle. The position vector of each particle ($\boldsymbol{p}_{\text{curr}}$) encodes a set of features, and the mapping of real to binary values, where 1 indicates the presence of the feature and 0 indicates its omission, is achieved by a threshold λ. The initialization of the different vectors was done as follows:

$$\boldsymbol{p}^0_{\text{curr}} = \boldsymbol{p}^0_{\text{best}} \sim \boldsymbol{U}(0,1), \tag{1}$$

$$\boldsymbol{v}^0 \sim \boldsymbol{U}(0,1), \tag{2}$$

$$\boldsymbol{g}^0_{\text{best}} = \underset{\text{AUC}}{\text{argmax}} \; \text{SE}\,(\text{particle}_{\boldsymbol{p}^0_{\text{curr}}}, \lambda) \;\; \forall\, \text{particle} \in \text{swarm} \tag{3}$$

where $\boldsymbol{U}(0,1)$ denotes a uniform distribution with values in the range $[0,1]$. The *Exemplar Assignment* algorithm used for selecting $\boldsymbol{p}_{\text{expl}}$ were similar to the one presented in [2] based on [8]. However, we introduced a hyperparameter η to regulate the learning probability, P_{c_i}, associated with each particle balancing the exploitation/exploration trade-off:

$$P_{c_i} = \eta \cdot \frac{(exp(10 \cdot (\text{rank}(i) - 1))/(\text{S - 1})}{exp(10) - 1} \tag{4}$$

where S is the population size, and $\text{rank}(i)$ is the ranking of the particle within the population determined by its fitness value.

[1]Hyperparameters: number of neighbors 5, euclidean distance, uniform neighbors weighting. The Scikit-Learn implementation was used [4].

On the other hand, two additional variations were introduced with respect to the original approach [2]. The first modification was the introduction of information about $\boldsymbol{g}_{\text{best}}$ when updating the velocity of each particle at iteration t. The second variation consisted in the use of a dynamic calculation of the particle inertia to promote the escape from local maxima. Therefore, the final equations used to update the position and velocity of each particle i across dimension d, were as follows:

$$v_{id}^{t+1} = dw \cdot v_{id}^{t} + c_1 \cdot U(0,1) \cdot (p_{\text{expl}_{id}}^{t} - p_{\text{curr}_{id}}^{t}) + c_2 \cdot U(0,1) \cdot (g_{\text{best}_{id}}^{t} - p_{\text{curr}_{id}}^{t}) \tag{5}$$

$$dw = (1-\gamma) \cdot w + \gamma \cdot exp(-|\boldsymbol{p}_{\text{curr}_{\boldsymbol{i}}} - \boldsymbol{p}_{\text{best}_{\boldsymbol{i}}}|) \tag{6}$$

$$x_{id}^{t+1} = x_{id}^{t} + v_{id}^{t+1} \tag{7}$$

being γ a hyperparameter that controls the weight given to the inertia, w, during the calculation of the dynamic inertia dw; and c_1 and c_2 acceleration constants. At this point it should be clarified that when the dimensionality of $\boldsymbol{g}_{\text{best}}^{\boldsymbol{t}}$ is less than $\boldsymbol{p}_{\text{curr}_{\boldsymbol{i}}}^{\boldsymbol{t}}$, the second part of the equation was selected to 0 for the extra dimensions of $\boldsymbol{g}_{\text{best}}^{\boldsymbol{t}}$.

Regarding particle dimensions, as in the the original approach [2], the particles were divided into uniform groups of different size L, determined by Equations 8 and 9:

$$L = N \cdot \frac{\text{Div}}{\text{NbrDiv}} \tag{8}$$

$$\text{DivSize} = \frac{\text{PopSize}}{\text{NbrDiv}} \tag{9}$$

being DivSize the number of particles in each division; PopSize the population size; NbrDiv the number of population divisions; Div the number of the division; N the number of features; and L the size of the particles in division Div.

This variable-length particle representation is biased towards the selection of variables that are encoded in the initial positions. To exploit this property, the input features are sorted according to an importance score [2]. Therefore, the most important variables will appear in the initial positions and are more represented. In this work, the ranking of the input features was defined by the absolute value of Pearson's correlation of the predictor variable with the dependent variable (considering the quantitative value).

Finally, this implementation also considers the hyperparameters α and β used to apply the *Exemplar Assignment* or *Length Changing* algorithms when there is no improvement in the fitness value of particle i for more than α iterations, or in $\boldsymbol{g}_{\text{best}}$ for more than β iterations, respectively [2].

Appendix 4

This appendix details all the hyperparameters of the algorithms used for amyloid status classification, and the input data on which they were applied. For all models, the Scikit-Learn [4] implementation was used except for the VLPSO strategy which used homemade software available on GitHub. For all except the tree-based models, the data were previously standardized to z-scores using the statistics from the training dataset.

Table A4: Model hyperparameters used to classify amyloid status based on neuropsychological and demographic variables, acoustic data (presented in Table A1), and combining acoustic variables with demographic information.

**Model**	**Hyperparameter**
PCA[1]-SVM	Radial basis kernel<br>$\gamma = (\text{Num. features} \cdot var(\boldsymbol{X}))^{-1}$<br>C = 0.75
PCA[1]-KNN	Neighbors = 5<br>Euclidean distance<br>Uniform neighborhood weights
VLPSO-KNN[2]	(VLPSO) Population size = 150<br>(VLPSO) Iterations = 500<br>(VLPSO) $\eta = 0.2$<br>(VLPSO) $w = 0.2$<br>(VLPSO) $\gamma = 0.5$<br>(VLPSO) $\lambda = 0.5$<br>(VLPSO) $c_1 = 0.1$<br>(VLPSO) $c_2 = 0.2$<br>(VLPSO) NbrDiv = 10<br>(VLPSO) $\alpha = 40$<br>(VLPSO) $\beta = 100$<br>(VLPSO) Random inits = 30<br>(KNN) Neighbors = 5<br>(KNN) Euclidean distance<br>(KNN) Uniform neighborhood weights
LR	L1 and L2 penalties
PCA[1]-LR	L2 penalty
PCA[1]-LR	L1 and L2 penalties
RF (LC)	500 estimators<br>Gini impurity<br>Max depth = 5

PCA: Principal Component Analysis; SVM: Support Support Vector Machine; KNN: K-Nearest Neighbors; VLPSO: Variable-Length Particle Swarm Optimization; LR: Logistic Regression; RF (LC/HC): Random Forest High capacity/Low capacity).

[1]Number of components tested from 5 to 30.

[2]Model not applied to the dataset based on neuropsychological and demographic variables.

Table A4: (continued)

Model	Hyperparameter
RF (LC)	Min samples leaf = 4<br>Max features = 0.5
RF (HC)	500 estimators<br>Gini impurity<br>Max depth = 10<br>Min samples leaf = 2<br>Max features = 0.75

PCA: Principal Component Analysis; SVM: Support Support Vector Machine; KNN: K-Nearest Neighbors; VLPSO: Variable-Length Particle Swarm Optimization; LR: Logistic Regression; RF (LC/HC): Random Forest High capacity/Low capacity).

[1]Number of components tested from 5 to 30.

References

[1] Florian Eyben, Klaus R Scherer, Björn W Schuller, Johan Sundberg, Elisabeth André, Carlos Busso, Laurence Y Devillers, Julien Epps, Petri Laukka, Shrikanth S Narayanan, et al. The geneva minimalistic acoustic parameter set (gemaps) for voice research and affective computing. *IEEE transactions on affective computing*, 7(2):190–202, 2015.

[2] Binh Tran, Bing Xue, and Mengjie Zhang. Variable-length particle swarm optimization for feature selection on high-dimensional classification. *IEEE Transactions on Evolutionary Computation*, 23(3):473–487, 2018.

[3] Rizgar Zebari, Adnan Abdulazeez, Diyar Zeebaree, Dilovan Zebari, and Jwan Saeed. A comprehensive review of dimensionality reduction techniques for feature selection and feature extraction. *Journal of Applied Science and Technology Trends*, 1(2):56–70, 2020.

[4] Lars Buitinck, Gilles Louppe, Mathieu Blondel, Fabian Pedregosa, Andreas Mueller, Olivier Grisel, Vlad Niculae, Peter Prettenhofer, Alexandre Gramfort, Jaques Grobler, et al. Api design for machine learning software: experiences from the scikit-learn project. *arXiv preprint arXiv:1309.0238*, 2013.

[5] Dongshu Wang, Dapei Tan, and Lei Liu. Particle swarm optimization algorithm: an overview. *Soft computing*, 22:387–408, 2018.

[6] James Kennedy and Russell Eberhart. Particle swarm optimization. In *Proceedings of ICNN'95-international conference on neural networks*, volume 4, pages 1942–1948. IEEE, 1995.

[7] Riccardo Poli, James Kennedy, and Tim Blackwell. Particle swarm optimization: An overview. *Swarm intelligence*, 1:33–57, 2007.

[8] Jing J Liang, A Kai Qin, Ponnuthurai N Suganthan, and S Baskar. Comprehensive learning particle swarm optimizer for global optimization of multimodal functions. *IEEE transactions on evolutionary computation*, 10(3):281–295, 2006.
